# AAV-Mediated Knock-Down of HRC Exacerbates Transverse Aorta Constriction-Induced Heart Failure

**DOI:** 10.1371/journal.pone.0043282

**Published:** 2012-08-28

**Authors:** Chang Sik Park, Hyeseon Cha, Eun Jeong Kwon, Dongtak Jeong, Roger J. Hajjar, Evangelia G. Kranias, Chunghee Cho, Woo Jin Park, Do Han Kim

**Affiliations:** 1 College of Life Sciences and Systems Biology Research Center, Gwangju Institute of Science and Technology (GIST), Buk-gu, Gwangju, Republic of Korea; 2 Cardiovascular Research Center, Mount Sinai School of Medicine, New York, New York, United States of America; 3 Department of Pharmacology & Cell Biophysics, College of Medicine, University of Cincinnati, Cincinnati, Ohio, United States of America; Medical University Innsbruck, Austria

## Abstract

**Background:**

Histidine-rich calcium binding protein (HRC) is located in the lumen of sarcoplasmic reticulum (SR) that binds to both triadin (TRN) and SERCA affecting Ca^2+^ cycling in the SR. Chronic overexpression of HRC that may disrupt intracellular Ca^2+^ homeostasis is implicated in pathogenesis of cardiac hypertrophy. Ablation of HRC showed relatively normal phenotypes under basal condition, but exhibited a significantly increased susceptibility to isoproterenol-induced cardiac hypertrophy. In the present study, we characterized the functions of HRC related to Ca^2+^ cycling and pathogenesis of cardiac hypertrophy using the in vitro siRNA- and the in vivo adeno-associated virus (AAV)-mediated HRC knock-down (KD) systems, respectively.

**Methodology/Principal Findings:**

AAV-mediated HRC-KD system was used with or without C57BL/6 mouse model of transverse aortic constriction-induced failing heart (TAC-FH) to examine whether HRC-KD could enhance cardiac function in failing heart (FH). Initially we expected that HRC-KD could elicit cardiac functional recovery in failing heart (FH), since predesigned siRNA-mediated HRC-KD enhanced Ca^2+^ cycling and increased activities of RyR2 and SERCA2 without change in SR Ca^2+^ load in neonatal rat ventricular cells (NRVCs) and HL-1 cells. However, AAV9-mediated HRC-KD in TAC-FH was associated with decreased fractional shortening and increased cardiac fibrosis compared with control. We found that phospho-RyR2, phospho-CaMKII, phospho-p38 MAPK, and phospho-PLB were significantly upregulated by HRC-KD in TAC-FH. A significantly increased level of cleaved caspase-3, a cardiac cell death marker was also found, consistent with the result of TUNEL assay.

**Conclusions/Significance:**

Increased Ca^2+^ leak and cytosolic Ca^2+^ concentration due to a partial KD of HRC could enhance activity of CaMKII and phosphorylation of p38 MAPK, causing the mitochondrial death pathway observed in TAC-FH. Our results present evidence that down-regulation of HRC could deteriorate cardiac function in TAC-FH through perturbed SR-mediated Ca^2+^ cycling.

## Introduction

The histidine-rich calcium binding protein (HRC), located in the luminal region of sarcoplasmic reticulum (SR), is a low-affinity and high-capacity Ca^2+^-binding protein [1], [2], [3]. The histidine- and glutamic acid-rich repeat region of HRC binds to the KEKE motif of the luminal region of triadin (TRN) [4], the site for binding to both calsequestrin (CSQ) [5], [6] and ryanodine receptor (RyR) [7]. The same region of HRC also interacts with the N-terminal cation transporter domain of SR Ca^2+^-ATPase (SERCA) in a Ca^2+^ concentration–dependent way [8]. However, the physiological importance of the multi-protein interactions between HRC and other proteins in the SR has remained to be clarified.

**Figure 1:**
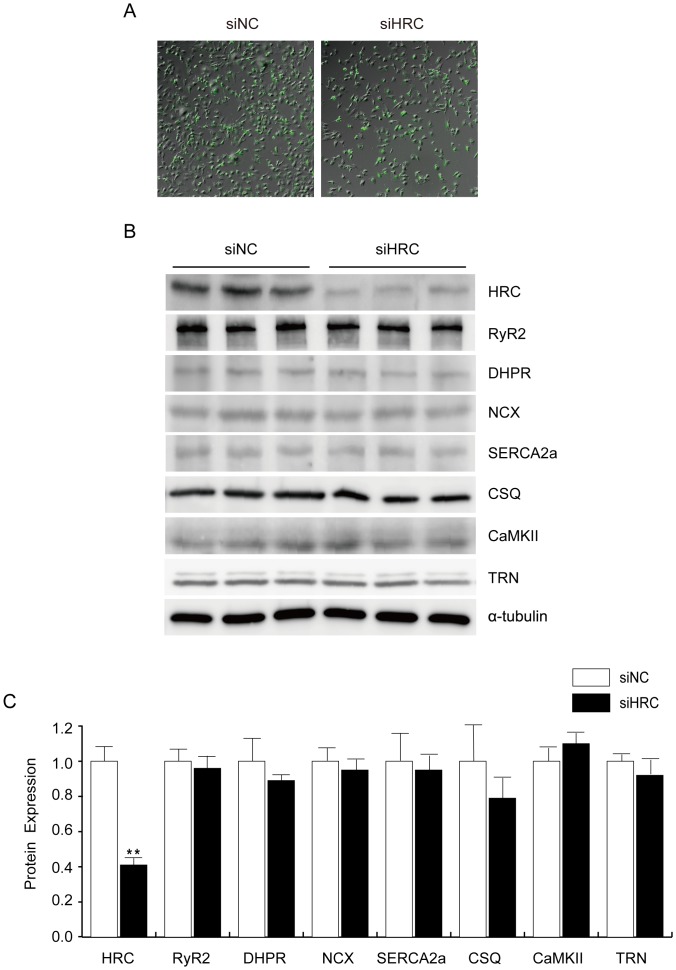
siRNA-mediated HRC knock-down (KD) and the expressional profiles of SR proteins in the control and KD samples. siNC and siHRC oligonucleotides (Dharmacon) were transfected to neonatal rat ventricular cells (NRVCs). After 48 h, transfected NRVCs were solubilized with 1% SDS lysis buffer, and western blot analyses were performed using various antibodies. A: Western blot result of SR proteins in NRVCs after HRC-KD. RyR, ryanodine receptor; HRC, histidine-rich calcium binding protein; SERCA2a, sarcoplasmic reticulum Ca^2+^ ATPase 2a; DHPR, dihydropyridine receptor; NCX, Na^+^-Ca^2+^ exchanger; CSQ, calsequestrin; CaMKII, Ca^2+^/calmodulin-dependent kinase; TRN, triadin. B: Relative expression levels of SR proteins after HRC-KD. siNC, negative control of knock-down oligonucleotide; siHRC, HRC-KD oligonucleotide (**P<0.01). Note that there were no expressional changes of other SR proteins by knock-down of HRC.

**Figure 2 pone-0043282-g002:**
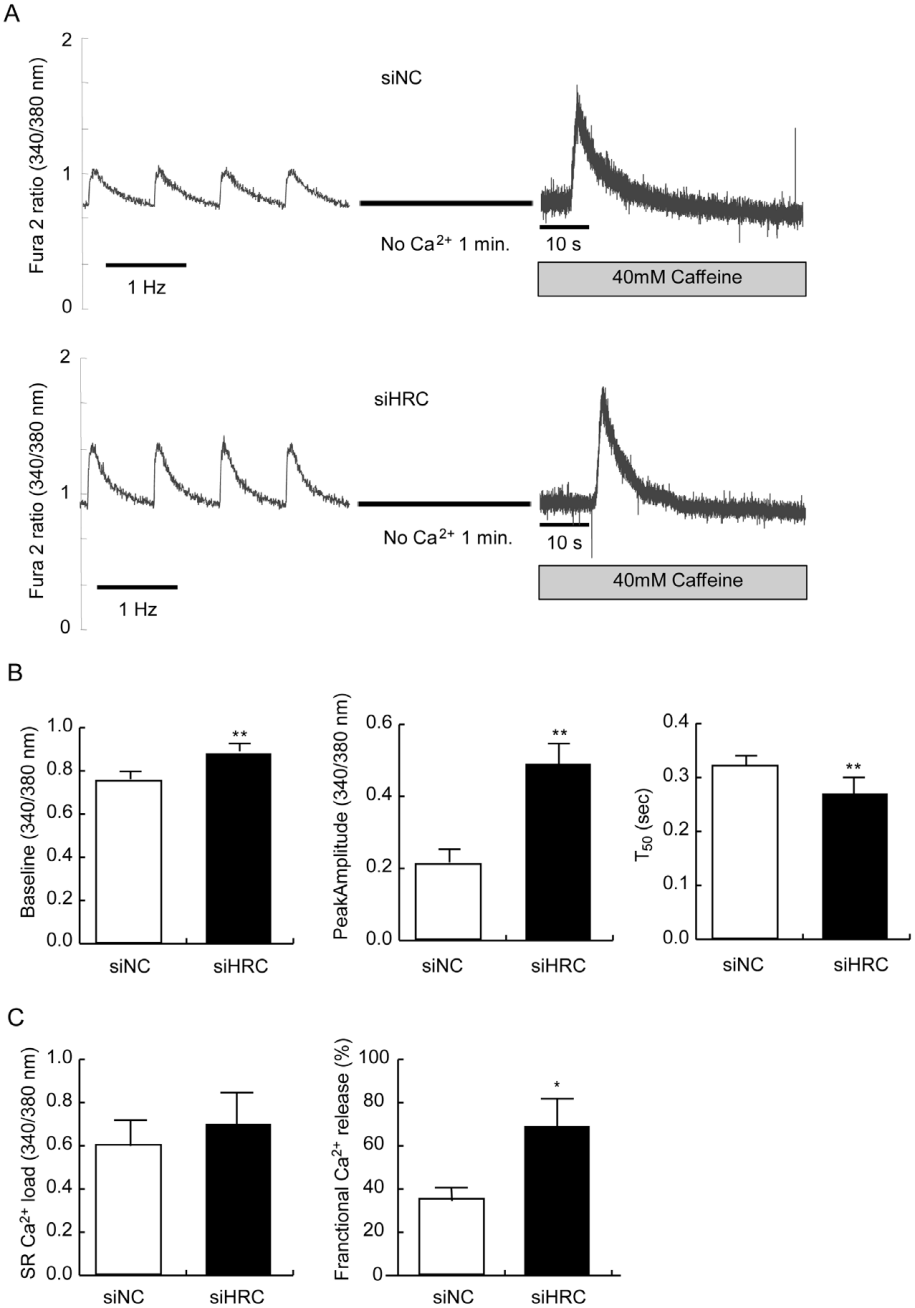
Electrical- and caffeine-induced Ca^2+^ transients in NRVCs. After 48 h of transfection with siNC or siHRC oligonucleotide, the NRVCs were treated with Fura 2-AM, and Ca^2+^ transients were measured at 1-Hz electrical stimulation or 40 mM caffeine application in Tyrode solution to measure sarcoplasmic reticulum (SR) Ca^2+^ load using IonOptix. A: Typical records of electrical- and caffeine-induced Ca^2+^ transients in siNC and siHRC oligonucleotide treated NRVCs. B: Significantly changed parameters of Ca^2+^ transients after HRC-KD. C: Left, the SR Ca^2+^ load was not significantly different between siNC and siHRC oligo-transfected NRVCs Right, the fractional Ca^2+^ release was significantly increased in HRC-KD NRVCs (siHRC). 16 sets of siNC and 21 sets of siHRC NRVCs were used for statistical analyses. Values are means ± S.E. *P<0.05.

We have previously reported that HRC overexpression increased SR Ca^2+^ load both in neonatal and adult rat cardiomyocytes [9]. In addition, adenovirus-mediated HRC overexpression in adult rat cardiomyocytes increased time to reach 50% relaxation (T_50_) and time constant of decay, and decreased peak amplitude of Ca^2+^-induced Ca^2+^ release, and fractional shortening [10]. Overexpression of HRC in transgenic mice resulted in impaired SR Ca^2+^ uptake rates and depressed cardiomyocyte Ca^2+^ transient decay, without significant changes in Ca^2+^ transient amplitude or SR Ca^2+^ load, indicating an inhibitory role of HRC for SERCA activity [11]. Furthermore, HRC transgenic mice expressed hypertrophic phenotypes developing increased heart weight/body weight ratio (HW/BW) and induction of fetal gene expression of atrial natriuretic factor (ANF) and β-myosin heavy chain (β-MHC) [11]. HRC knock-out (KO) mice showed relatively normal phenotypes under no stressful conditions, but exhibited a significantly increased susceptibility to isoproterenol (ISO)-induced cardiac hypertrophy suggesting a regulatory role of HRC in the cardiac remodeling [12]. Collectively, HRC may be an important Ca^2+^ cycling regulator in SR of which expression could be associated with pathogenesis of the heart. However, the precise mechanism of HRC mediated inhibition of Ca^2+^ cycling and the long term cardiac remodeling has remained to be clarified.

The present study was designed on the basis of the hypothesis that HRC knock-down (KD) enhances Ca^2+^ cycling and cardiac function through the increased activity of SERCA2 and RyR2. Thus, we used synthetic siRNA oligonucleotides and adeno-associated virus (AAV) to knock-down HRC expression *in vitro* (for short term effect) and *in vivo* (for chronic effect), respectively. HRC-KD in neonatal rat ventricular cells (NRVCs) or HL-1 cells showed enhanced Ca^2+^ cycling, but the resting Ca^2+^ concentration was increased due possibly to Ca^2+^ leak through the activated RyR2. HRC-KD using AAV9-shHRC resulted in more decreased cardiac function, and increased cardiac fibrosis and apoptosis causing more severe heart failure in mice under pressure-overload by transverse aortic constriction (TAC). Our concomitant biochemical study showed that the increased Ca^2+^-leak and elevated cytosolic Ca^2+^ due to HRC-KD could enhance phosphorylation of CaMKII - p38 MAPK pathway resulting in the increased apoptosis and heart failure. Collectively, the present study suggests that HRC is an important regulator of Ca^2+^ homeostasis which is essential for normal functions of the heart.

**Figure 3 pone-0043282-g003:**
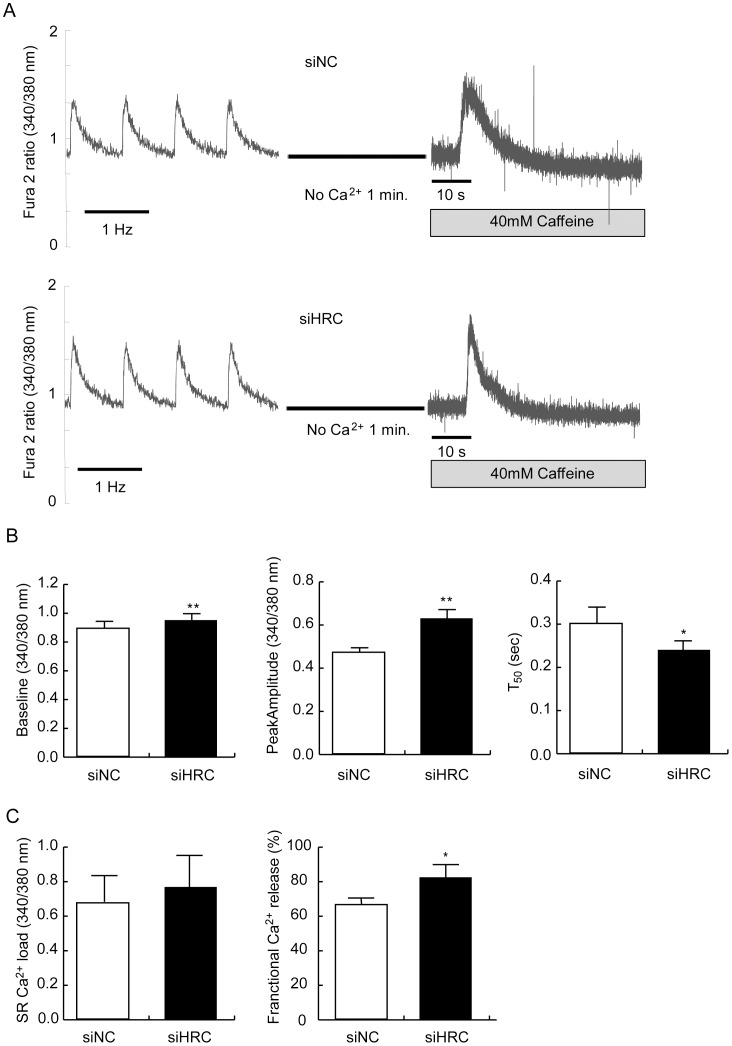
Electrical- and caffeine-induced Ca^2+^ transients in NRVCs treated with ISO. After 48 h of transfection with siNC or siHRC oligonucleotide, the NRVCs were treated with Fura 2-AM, Ca^2+^ transients were measured at 1-Hz electrical stimulation at 1 µmol/L isoproterenol (ISO) using IonOptix. A: Typical records of Ca^2+^ transients at 1 µmol/L ISO in siNC and siHRC oligonucleotide treated NRVCs. B: Significantly changed parameters of Ca^2+^ transients after HRC-KD. Baseline, resting cytosolic Ca^2+^ concentration; peak amplitude, the amount of Ca^2+^ released from SR; T_50_, time to 50% baseline fluorescence; fractional Ca^2+^ release, depolarization-induced Ca^2+^ release/caffeine-induced Ca^2+^ release (*P<0.05).

## Results

### Successful (KD) of HRC in NRVCs

The chronic HRC overexpression or KO studies presented higher expression of TRN protein [10], [12]. The phenotypic changes occurring in response to overexpression or ablation of HRC could be associated with an adaptive developmental or compensatory response system-wide [13]. Therefore, in this study, an acute partial KD of HRC was performed in NRVCs using siRNA oligonucleotides targeted to HRC to evaluate the effects of decreased HRC expression on Ca^2+^ cycling without possible change in protein expression related to Ca^2+^ cycling. The siRNA oligonucleotides were successfully transfected to NRVCs (the calculated transfection efficiency was more than 99%) (Figure 1A) and the cells showed a 70% reduction of HRC protein expression, where was no noticeable change of protein expression in RyR2, DHPR, NCX, SERCA2, CSQ, CaMKII, or TRN (Figure 1). We also conducted the above experiments using HL-1 cells originally derived from mouse atrial cells. The results are similar between the different cell types (Figure S1).

**Figure 4:**
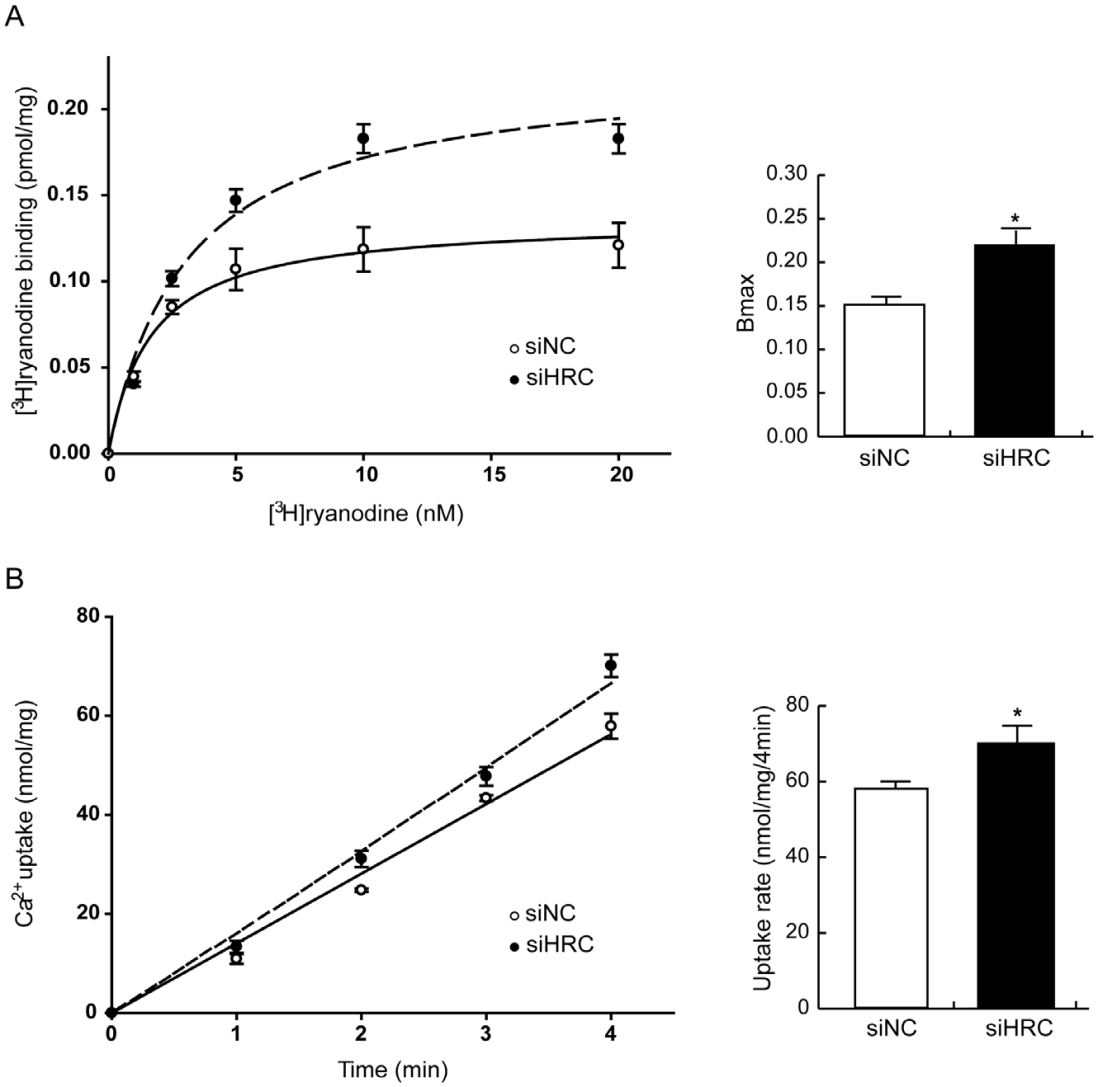
[^3^H]-ryanodine binding and oxalate-supported Ca^2+^ uptake assays. After 48 h of siRNA transfection, the NRVCs were used for [^3^H]-ryanodine binding and oxalate-supported Ca^2+^ uptake assays. A: [^3^H]-ryanodine binding assay results using siNC and siHRC transfected NRVCs. The significantly different B_max_ values of [^3^H]-ryanodine binding were 0.146±0.011 and 0.224±0.03 pmol/mg for siNC (○) and siHRC (•) samples, respectively. The Kd values are not significantly different between the 2 samples (siNC: 2.786±0.664 nM vs. siHRC: 3.052±0.702 nM). B: Oxalate-supported Ca^2+^ uptake assay results. Oxalate-supported SR-based Ca^2+^ uptake in siNC (○) and siHRC (•) transfected NRVCs were determined at 1, 2, 3 and 4 min. The rates of Ca^2+^ uptake were 57.89±2.54 and 70.11±2.26 nmol/mg/4 min for siNC and siHRC samples, respectively. 7 sets of siNC and siHRC NRVCs were used for statistical analyses (*P value <0.05).

### Enhanced Ca^2+^ Cycling in NRVCs with No Change of SR Ca^2+^ Load

We measured typical traces of 1-Hz electrical stimulation–induced Ca^2+^ transients in siNC and siHRC oligo transfected NRVCs to investigate whether HRC-KD affected Ca^2+^ transients in these cells under basal and isoproterenol (ISO) treated conditions (Figures 2 and 3). Under basal condition, compared with siNC NRVCs (n = 16), the resting cytosolic Ca^2+^ concentration (baseline) was significantly increased in HRC-KD NRVCs (n = 33) (siNC: 0.77±0.06 vs. siHRC: 0.88±0.03 arbitrary unit; *P*<0.05), and the peak amplitude of the Ca^2+^ transients was also increased in HRC-KD cells (siNC: 0.21±0.04 vs. siHRC: 0.49±0.07; *P*<0.05), suggesting increased Ca^2+^ release through RyR2. The time to reach 50% baseline (T_50_ baseline), on the other hand, was significantly reduced (siNC: 0.32±0.02 s vs. siHRC: 0.27±0.04 s; *P*<0.05), suggesting faster Ca^2+^ uptake through SERCA2 (Figure 2B). Under ISO treated condition, compared with siNC NRVCs (n = 27), the resting cytosolic Ca^2+^ concentration (baseline) was again significantly increased in HRC-KD NRVCs (n = 25) (siNC: 0.89±0.06 vs. siHRC: 0.95±0.04 arbitrary unit; *P*<0.05), and the peak amplitude of Ca^2+^ transients was also increased in HRC-KD cells (siNC: 0.47±0.01 vs. siHRC: 0.63±0.03; *P*<0.05), suggesting increased Ca^2+^ release through RyR2. The time to reach 50% baseline (T_50_ baseline), on the other hand, was significantly reduced (siNC: 0.30±0.08 s vs. siHRC: 0.24±0.04 s; *P*<0.05), suggesting faster Ca^2+^ uptake through SERCA2 (Figure 3). HRC-KD in HL-1 cells also showed similar enhanced Ca^2+^ cycling (Figure S2B). Previous reports have suggested that HRC could interact with TRN and SERCA [4], [8], and TRN affected RyR2 through a direct interaction with the channel [14], [15], [16]. Adenovirus-mediated HRC overexpression in adult rat cardiomyocytes showed increased TRN and junctin (JTN) expression, and its inhibitory effect on RyR2 and SERCA2 [10], consistent with our results (Figure 2).

**Figure 5:**
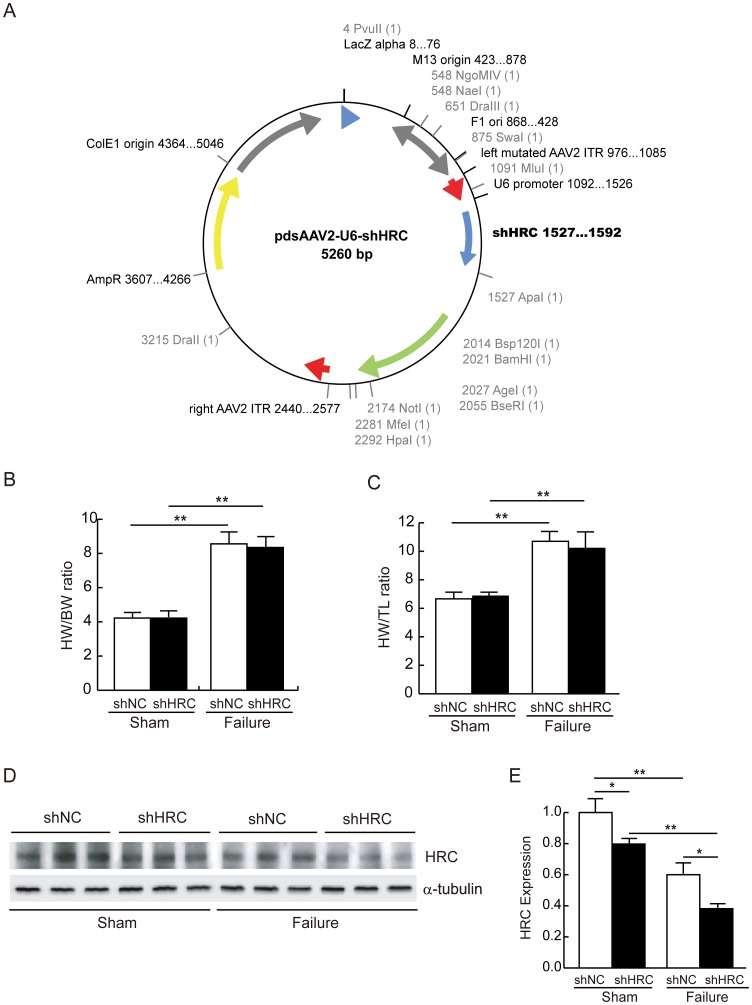
AAV9 virus -mediated HRC-KD in sham and TAC-FH samples. A: Schematic figure for the construction of shNC- and shHRC-AAV9 viruses. B: Heart weight per body weight (HW/BW) ratio after 11 weeks of TAC operation. C: Heart weight per tibia length (HW/TL) ratio after 11 weeks of TAC operation. D: Western blot result of HRC expression from 4 groups, shNC sham, shHRC sham, shNC failure and shHRC failure. E: Summarized data for HRC expression (*P<0.05, **P<0.01). Note that the HRC expression was down-regulated both in sham and failure samples.

We also measured 20 mM caffeine-induced Ca^2+^ transients in siNC and siHRC NRVCs under basal and ISO conditions to examine whether HRC-KD affected SR Ca^2+^ load in the cells. There was no significant change of caffeine-induced Ca^2+^ transients between siNC and siHRC NRVCs (Basal, siNC : 0.702±0.054, n = 7 vs. siHRC : 0.605±0.079, n = 7; ISO, siNC : 0.67±0.15, n = 7 vs. siHRC : 0.77±0.14, n = 7) (Figures 2C and 3C). However, fractional Ca^2+^ release (amplitude of electrically-evoked Ca^2+^ transient/amplitude of caffeine-induced Ca^2+^ transient) [10] was significantly increased in HRC-KD NRVCs under both basal and ISO conditions indicating that more Ca^2+^ was released from the SR upon electrical stimulation in HRC-KD cells (Basal, siNC: 35.08±5.39 vs. siHRC: 69.09±12.21%; ISO, siNC: 66.32±6.95 vs. siHRC: 82.42±7.57%) (Figures 2C and 3C). The less difference of fractional Ca^2+^ release between siNC and siHRC under ISO condition than that under basal condition (16.1 vs. 34.0) could be due to fact that Ca^2+^ release was already activated under ISO condition in siNC cells. HL-1 cells showed no significant change of caffeine-induced Ca^2+^ transients, but significantly enhanced fractional Ca^2+^ release under basal condition, similar to NRVCs (Figure S2C).

**Figure 6:**
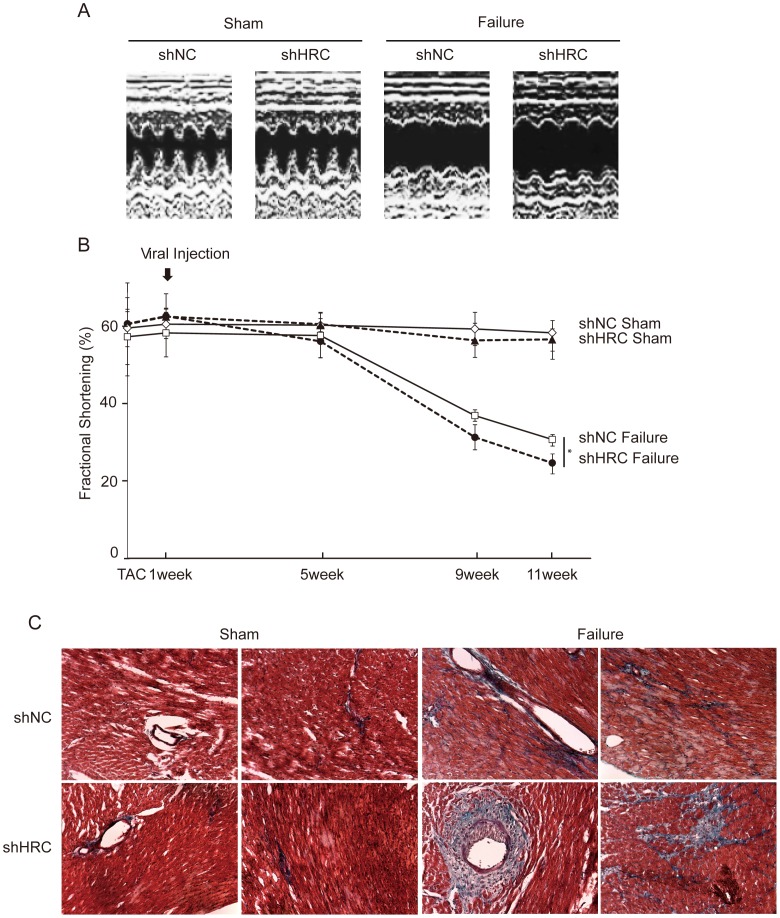
Severely down-regulated cardiac function and cardiac fibrosis in HRC-KD heart under heart failure condition. A: Representative M-mode echocardiograms of all groups. Echocardiography on the experimental animals was performed at 1, 5, 9, and 11 weeks after sham or TAC operation. B: Changes in fractional shortening after viral injection for the duration of 11 weeks in sham- and TAC-operated shNC and shHRC mice. HRC-KD heart showed most severely down-regulated fractional shortening (FS) under heart failure condition. C: Masson’s trichrome staining results. Blue color indicates the appearance of collagen; red color indicates that the cytoplasmic area is occupied by myocytes and erythrocytes; black color indicates the presence of nuclei. HRC-KD heart showed most severe fibrosis under heart failure condition. shNC, negative control of AAV9; shHRC, HRC-KD through AAV9 (*P<0.05).

**Table 1:** Echocardiographic data associated with HRC-KD in TAC-HF model.

	shNC		shHRC	
	Sham (n = 4)	Failure (n = 7)	Sham (n = 4)	Failure (n = 7)
SWTd (mm)	0.875±0.05	0.971±0.125	0.85±0.058	0.85±0.238
LVEDD (mm)	3.475±0.287	4.0±0.208*	3.3±0.283	4.45±0.311##,†
PWTd (mm)	0.9±0.141	0.986±0.09	0.95±0.058	0.8±0.082#,†
SWTs (mm)	1.725±0.222	1.486±0.146*	1.65±0.252	1.325±0.096#,†
LVESD (mm)	1.4±0.216	2.871±0.386**	1.3±0.183	3.225±0.457##,†
PWTs (mm)	1.825±0.206	1.486±0.168*	1.725±0.206	1.25±0.1##,†
FS (%)	59.5±4.359	31.0±2.757**	57.75±4.5	25.25±3.304##,†

Parameters of echocardiography obtained from shNC and shHRC mice at 11 weeks after sham or TAC operation. The most severe cardiac dilation and fractional shortening (FS) were observed in shHRC failure mice. shNC, negative control hearts through AAV9; shHRC, HRC-KD hearts through AAV9. SWTd, diastolic septal wall thickness; SWTs, septal wall thickness; LVEDD, LV end-diastolic dimension; LVESD, LV end-systolic dimension; PWTd, diastolic posterior wall thickness; PWTs, systolic posterior wall thickness; FS, fractional shortening.

* , *P*<0.05;

** , *P*<0.01 versus shNC sham;

# , *P*<0.05;

## , *P*<0.01 versus shHRC sham;

† , *P*<0.05;

†† , *P*<0.01 versus shNC failure.

### Increased RyR2 or SERCA2 Activity in HRC-KD NRVCs

To examine the underlying mechanisms for the enhanced Ca^2+^ cycling in HRC-KD NRVCs (Figures 2 and 3), [^3^H]-ryanodine binding and oxalate-supported Ca^2+^ uptake assays were performed. The maximum [^3^H]-ryanodine binding (B_max_) was significantly increased in siHRC cells (n = 27), compared with siNC cells (n = 17) (siNC: 0.146±0.011 pmol/mg vs. siHRC: 0.224±0.03 pmol/mg; *P*<0.01) without a significant change in dissociation constant of [^3^H]-ryanodine binding (Kd) (siNC: 2.786±0.664 nM vs. siHRC: 3.052±0.702) (Figure 4A). In previous reports, it was suggested that [^3^H]-ryanodine binding was a useful method to measure RyR2 activity, because ryanodine could bind to only the open state of RyR2 and hence [^3^H]-ryanodine binding can reflect the open state of the channel [17], [18]. Our ryanodine binding result suggests that HRC-KD increases the open-state of RyR2 without noticeable change of ryanodine binding affinity.

**Figure 7:**
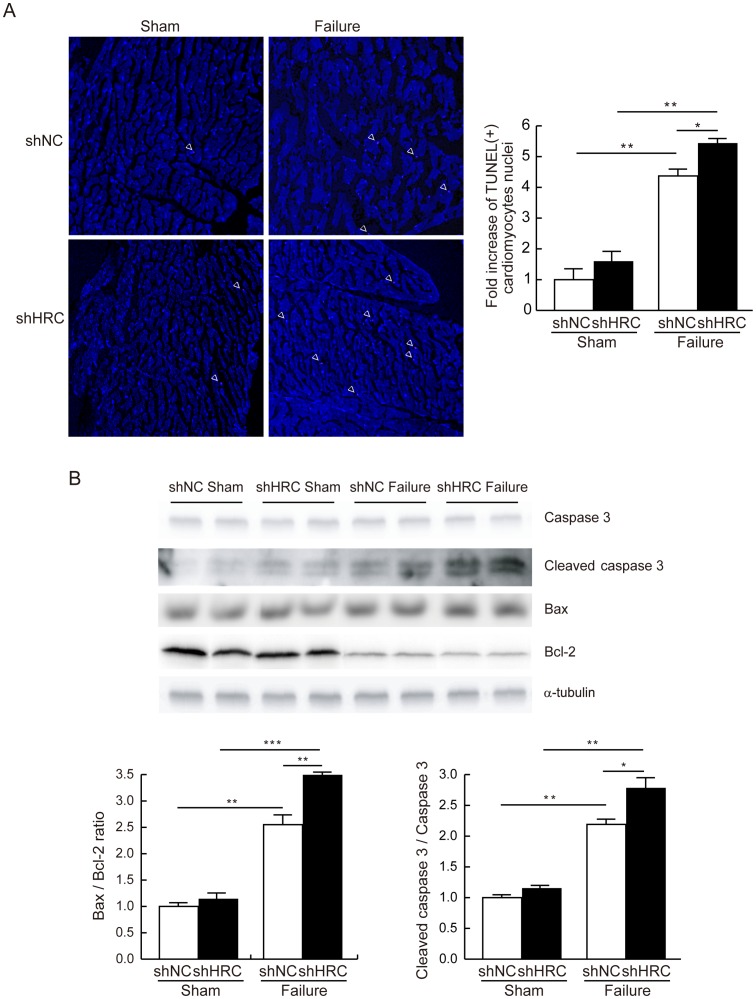
Increased apoptosis in HRC-KD heart under TAC-HF. TUNEL assay was performed using paraffin-embedded heart sections. A: Results of TUNEL assay. Left, white arrow heads show TUNEL positive pink signal. Right, relative numbers of TUNEL positive cardiomyocyte nuclei (shNC sham: 1±0.46; shNC failure: 4.38±0.21; shHRC sham: 1.6±0.4; shHRC failure: 5.44±0.17). B: Upper, western blot results of caspase 3, cleaved caspase 3, Bax and Bcl-2 from shNC sham, shNC failure, shHRC sham, and shHRC failure mouse hearts. Lower, summary of Bax per Bcl-2 ratio and the expressional changes of the apoptosis marker, cleaved caspase 3 (*P<0.05, **P<0.01).

The SR Ca^2+^ uptake rate was highly increased in siHRC cells (n = 7) compared with siNC cells (n = 5) (siNC: 57.89±2.54 nmol/mg/4 min vs. siHRC: 70.11±2.26 nmol/mg/4 min; *P*<0.05) (Figure 4B), suggestive of increased SERCA2 activity. HRC-KD in HL-1 cells also showed enhanced activities of RyR2 and SERCA2 (Figure S3). These results further indicate that the enhanced Ca^2+^ cycling in HRC-KD cells is caused by the increased activities of RyR2 and SERCA2.

**Figure 8:**
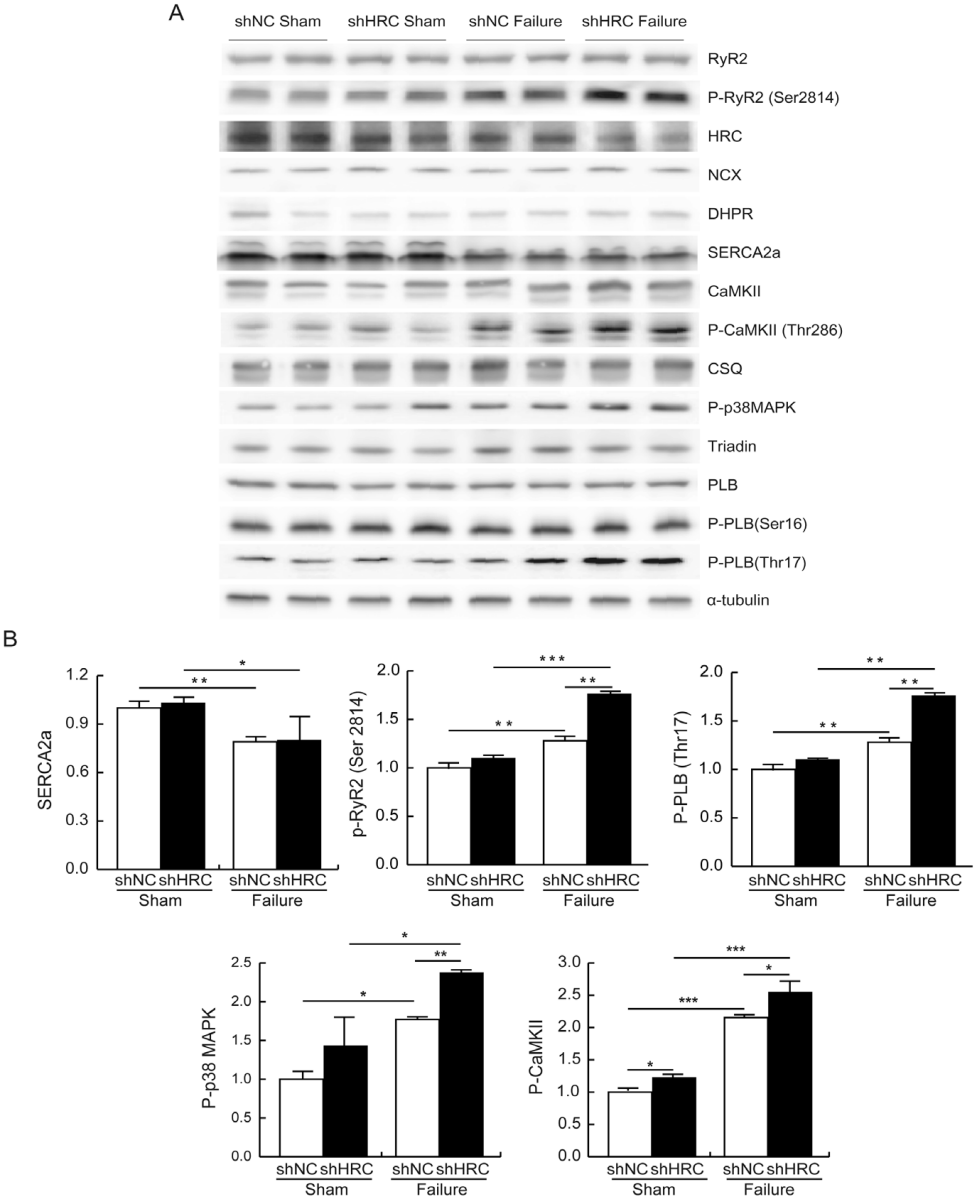
Investigation of the signaling cascade responsible for the enhanced apoptosis by HRC-KD in TAC-HF model. A: Western blot assay results. Heart lysates from shNC sham, shNC failure, shHRC sham, and shHRC failure mouse hearts were probed with antibodies against various target proteins involved in the apoptosis signaling pathway and Ca^2+^ cycling. P-CaMKII, phosphorylated CaMKII; P-p38 MAPK, phosphorylated p38 MAPK; PLB, phospholamban; P-PLB, phosphorylated phospholamban. B: Relative expression of differentially expressed proteins in shNC sham, shNC failure, shHRC sham, and shHRC failure heart samples (*P<0.05, **P<0.01, ***P<0.001).

**Figure 9:**
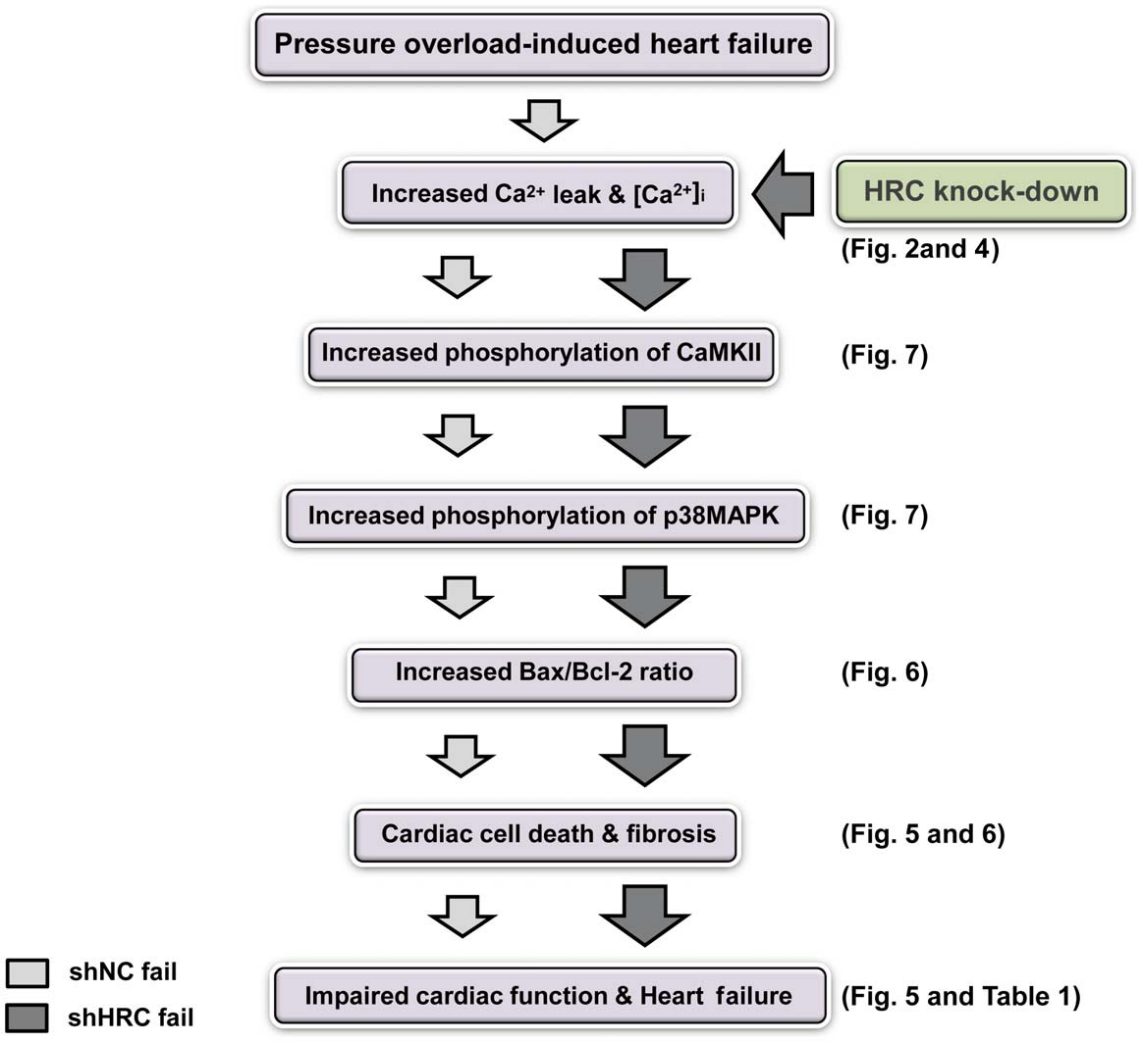
A flow-chart showing the putative mechanism for the deteriorated cardiac function by HRC-KD in TAC-HF model. Down-regulation of HRC expression induces increased RyR2 and SERCA2 activities followed by an increase of cytosolic Ca^2+^, which leads to activation of CaMKII and increased mitochondrial Ca^2+^ uptake. The activated CaMKII phosphorylates downstream kinases and causes cardiac cell death through an imbalance of the Bax/Bcl-2 ratio.

### AAV-mediated KD of HRC could Exacerbate the Heart Function in TAC-FH

We first attempted to use AAV serotype 9 for cardiac-specific gene transfer and tagged DsRed as a fluorescent infection marker to investigate whether KD of HRC could improve the heart function in TAC-FH model, since HRC-KD in NRVCs and HL-1 cells showed enhanced Ca^2+^ cycling (Figure 2 and Figure S3). However, KD of HRC was failed with the recombinant condition, due possibly to the inhibition by the fluorescent molecule, DsRed (19). Removal of DsRed in the construct significantly improved the HRC-KD efficiency (Figure 5A). Since DsRed was removed for enhancing KD efficiency, we used AAV-GFP system instead to examine the transduction efficiency of AAV system indirectly (Figure S4). The calculated transfection efficiency was approximately 30%. After 11 weeks of TAC operation, heart weight over body weight (HW/BW) ratio and heart weight over tibia length (HW/TL) ratio were significantly increased in both shNC and shHRC mice. However, there were no statistically significant differences in HW/BW and HW/TL between shNC and shHRC mice (Figures 5B and 5C), indicating that heart failure was successfully induced by TAC in both cases. The results of KD showed that the expression of HRC was reduced 20% in the shHRC sham (n = 4) compared with shNC sham (n = 4), and 36% in the shHRC failure (n = 7) compared with shNC failure (n = 7) *in vivo* (Figure 5E). It is interesting to note that the expression level of HRC was significantly decreased by TAC regardless of the animal models (shNC and shHRC), suggesting that HRC expression is associated with pathogenesis of the heart.

The partially HRC-KD mice and age-matched control samples in sham or TAC were subjected to echocardiography. The results showed that HRC-KD led to further reduction in fractional shortening (FS) and further increase in dilation of the heart at 11 weeks post operation in the failure group (Figures 6A and 6B). HRC-KD also showed a further decrease of posterior wall thickness (PWT) and septal wall thickness (SWT) in the heart at 11 weeks post operation (Table 1), indicative of severe heart failure in HRC-KD hearts. However, there was no significant difference in echocardiographic parameters between shNC sham and shHRC sham. We further performed trichrome staining to determine the degree of fibrosis in the different heart samples. There were no visible signs of fibrosis in the shNC sham and the shHRC sham, but the shHRC failure group showed more severe fibrosis compared with shNC failure group (Figure 6C). These results suggest that HRC-KD alone did not induce cardiac dysfunction under normal conditions, but it could worsen cardiac dysfunction in heart failure conditions.

### AAV-mediated KD of HRC Increased Cardiac Cell Death

It has been reported that apoptosis is the critical mechanism present in hypertensive heart disease [20], and cardiac apoptosis leads to decreased numbers of cardiomyocytes, with dead cells being replaced by fibrous tissues [21]. Since there was severe fibrosis in the shHRC failure group (Figure 6C), we examined cardiac apoptosis by the TUNEL assay using paraffin-embedded heart tissues. TUNEL-positive cardiomyocyte nuclei were most abundant in shHRC failure hearts (n = 5) compared with shHRC sham (n = 5), shNC failure (n = 4) and shNC sham (n = 4) (Figure 7A). Then, we checked the expression levels of proteins related to cell death. The largest expression of cleaved caspase-3, an apoptosis marker protein, was most abundant and Bax/Bcl-2 ratio was also increased in shHRC failure hearts (Figure 7B), indicating that there was significantly increased cardiac apoptosis in the shHRC failure group causing the severe cardiac fibrosis. Thus, we further investigated the signaling mechanism of HRC-KD induced cardiac apoptosis and severe fibrosis in failing hearts.

### AAV-mediated KD of HRC Activates CaMKII - p38 MAPK Signaling Pathway

CaMKII, a common intermediate of various death signal-induced apoptotic pathways in cardiac cells [22], has been highly associated with the transition from pressure overload–induced cardiac hypertrophy to heart failure in mice [23], [24], [25], [26]. We investigated whether HRC-KD could induce CaMKII-mediated cardiac apoptosis by examining phosphorylation of CaMKII and its substrates, since HRC-KD increased cytosolic Ca^2+^ concentration in NRVCs and HL-1 cells (Figure 2 and Figure S2). The results showed that phosphorylation of CaMKII increased 2.8 fold in shHRC failure, but increased only 1.2 fold in shHRC sham hearts. In addition, phosphorylation levels of phospholamban (PLB), RyR2, and p38 MAPK were also significantly increased, although total protein expressions of PLB, RyR2, CaMKII and p38 MAPK were not significantly changed in shHRC failure (Figure 8). Phosphorylation of RyR2 and PLB may affect SR Ca^2+^ cycling, possibly resulting in Ca^2+^ leak [27], [28], [29]. The p38 MAPK pathway, downstream of CaMKII [30], was also significantly activated. However, the expression levels of other Ca^2+^ cycling proteins were not changed, except for SERCA2a, which was down-regulated approximately 30%. Therefore, HRC-KD could further affect the mitochondrial death pathway by causing an imbalance between the expression of Bax and Bcl-2 [30], [31]. Taken together, these results suggest that HRC-KD induces cardiac apoptosis through activation of CaMKII/p38 MAPK signaling pathways, causing deterioration of heart function under the heart failure conditions (Figure 9).

## Discussion

According to the previous reports, HRC could interact with TRN and SERCA and regulate Ca^2+^ cycling through inhibiting SERCA and RyR2 [4], [8], [9], [10]. Chronic overexpression of HRC in mice resulted in increased TRN expression and inhibition of SERCA2 with hypertrophic phenotypes of increased fetal gene expression, HW/BW ratio, and fibrosis generation, indicative of a deterioration of cardiac function [11]. On the other hand, HRC overexpression could have also beneficial effects such as protection of the heart from ischemia/reperfusion [32]. A previous HRC-KO study showed that ablation of HRC could not alter the Ca^2+^ cycling properties, but on the other hand it could increase TRN protein expression [12]. The phenotypic changes at protein or functional levels occurring in response to overexpression or ablation of HRC could largely be associated with the adaptive or compensatory remodeling system-wide changes depending on the degree of expression of HRC [13]. The present study of HRC-KD consists of mainly two parts, 1) in vitro use of siRNA to knock down HRC in NRVCs and HL-1 cells to examine the direct effect of HRC on SERCA2 and RyR2 (Figures 1–4, and Supplemental Figures 1–3), and 2) in vivo use of adeno-associated virus (AAV) to knock-down HRC in the diseased heart to examine any beneficial effect. We generated an AAV-mediated partial HRC-KD system targeted to mouse heart to explore the physiological role of HRC in the heart to minimize the secondary effects of other proteins up- or down-regulated during the remodeling processes of the gene targeted animals (Figure 1 and Figure S1). Our studies of HRC using the partial KD systems appears to be beneficial and provided several novel findings as follows: 1) Both RyR2 and SERCA activities were enhanced by KD of HRC (Figures 1, 4 and Figure S3), 2) The increased RyR2 activity could directly cause the increased resting and peak Ca^2+^ concentrations (Figures 2, 3 and S2), 3)) Heart failure (HF) induced by TAC was further exacerbated by HRC-KD (Figure 6) and 4) The exacerbated HF by HRC-KD is due at lease in part to the cytosolic Ca^2+^-leak and enhanced mitochondrial death pathways (Figures 7 and 8). Collectively, the present study suggests that the precise control of the expressional level of HRC is essential for keeping the normal functions of the heart.

### TAC-associated Deterioration of Cardiac Function in HRC-KD Mice

Since HRC-KD showed increased Ca^2+^ transient amplitude and enhanced activities of RyR2 and SERCA2, suggestive of an enhanced Ca^2+^ cycling phenotype (Figures 2–4 and Figures S2–S3) in both neonatal rat ventricular cells (NRVCs) and HL-1 cells, we expected that HRC-KD would enhance cardiac function and may halt remodeling under pathological conditions such as the hypertensive hypertrophy model. However, HRC-KD alone did not rescue or decelerate cardiac function. On the contrary, it was associated with severe ventricular dilation and significantly decreased fractional shortening, indicative of further deterioration of cardiac function in the TAC model (Figures 6A and 6B). The partial HRC-KD also resulted in cardiac fibrosis (Figure 6C) and increased rates of cardiac cell death under the heart failure condition (Figure 7) potentially causing severe cardiac fibrosis [21]. The HRC expression was significantly reduced in the control (shNC) heart failure condition (Figure 5D), similar to the previous finding [10]. The additional down-regulation of HRC mediated by AAV9-shHRC showed further deterioration of cardiac function, even though HRC-KD was partial, suggesting that a proper expression of HRC could be a pivotally important for keeping the heart in healthy conditions.

### Activation of CaMKII-p38 MAPK Pathway and Increased Cardiac Cell Death in HRC-KD Hearts

Although the Ca^2+^ cycling is enhanced by HRC-KD, the possible predominant effect of HRC on RyR2 as compared with the effect on SERCA could result in the increase in the cytosolic Ca^2+^ concentration. Increases in cytosolic Ca^2+^ concentration have been reported to induce activation of CaMKII, which is related to the transition from pressure overload–mediated hypertrophy to heart failure in mice [26], [29]. In addition, the expression level and activity of CaMKII are highly up-regulated in heart failure [23], [24], [33], [34]. In this study, we observed that the phosphorylation of CaMKII and p38 MAPK was highly increased in both heart failure groups, shNC failure and shHRC failure, mediated by sustained pressure overload; however, the phosphorylation of CaMKII was more extensive in the shHRC failure group than it was in the shNC failure group, suggesting the increased activity of CaMKII in HRC-KD hearts (Figure 8). We also observed that the phosphorylation of CaMKII was slightly, but significantly, increased in shHRC sham compared with shNC sham (1±0.05 in shNC sham vs. 1.22±0.18 in shHRC sham) and there was a tendency to increase the phosphorylation level of PLB, RyR2 and p38 MAPK, however, it was not statistically significant. This might be caused by insufficient increase of CaMKII phosphorylation in shHRC sham heart. CaMKII is known to activate p38 MAPK [35], which induces mitochondrial death signaling by creating an imbalance between the expression of Bax and Bcl-2 (pro- and anti-apoptotic proteins, respectively) [30], [31]. We also found that phosphorylation level of p38 MAPK was increased in the shHRC failure group compared with shNC failure group (Figure 8), which could induce an imbalance between Bax and Bcl-2 expression and lead to increased apoptosis in the hearts (Figure 7).

### HRC as an Important Regulator for Cardiac Function

The previous HRC-KO mice showed impaired weight gain and TRN overexpression [12]. Furthermore, this animal model was susceptible to isoproterenol (ISO)-induced cardiac hypertrophy. These increased hypertrophic responses under conditions of cardiac stress are consistent with a regulatory role for HRC in SR Ca^2+^ cycling *in vivo*. Thus, alterations in HRC levels, combined with additional genetic or environmental factors, may contribute to pathological hypertrophy and heart failure. Recently, a genetic variant of HRC–Ser96Ala–showed a significant correlation with ventricular tachycardia and sudden cardiac death in patients with idiopathic dilated cardiomyopathy [36], [37]. Furthermore, reductions in HRC expression level may interrupt intracellular Ca^2+^ homeostasis, leading to the development of heart failure [10]. On the other hand, overexpression of HRC could protect against ischemia/reperfusion induced cardiac injury [32]. Taken together, maintenance of HRC expression in the heart properly is important for maintaining the cardiac function, as HRC acts as an important regulator for cardiac performance. It will be interesting to see whether AAV-mediated HRC over-expression could restore cardiac function in the failed heart, since the expression level of HRC is substantially down-regulated in the TAC animals (Figure 5).

In conclusion, HRC plays an important role in the regulation of Ca^2+^ cycling, including SR Ca^2+^ uptake and release, through the direct interaction with SERCA and TRN, respectively. It may also have a role in the progression of heart failure under pathophysiological conditions through stress- induced apoptotic pathways including CaMKII and p38 MAPK (Figure 9). Development of a method to maintain the proper expression level of HRC will likely be crucial for management of the heart diseases in the future.

## Materials and Methods

### Ethics Statement

All animal experiments were approved by the Gwangju Institute of Science and Technology Animal Care and Use Committee (the permit number: GIST-2011-1).

### HL-1 Cell Culture

HL-1 cells obtained as a kind gift from Dr. W. Claycomb (Louisiana State University Medical Center) were maintained as described previously [38]. Briefly, cells were cultured on gelatin (0.02%, w/v)/fibronectin (10 µg/ml)-coated cell culture plates. The cells were maintained in Claycomb medium (SAFC BIOSCIENCES™) supplemented with 10% fetal bovine serum (Sigma-Aldrich Co.), 2 mM L-glutamine, 0.1 mM norepinephrine, 100 unit/ml penicillin, and 100 µg/ml streptomycin (Invitrogen). The culture medium was changed with fresh medium every 24 hours. The cells were grown at 37°C in an atmosphere of 5% CO_2_ and 95% air in an incubator.

### Primary Cell Culture and siRNA oligo Transfection

Primary cultures of neonatal rat ventricular cells (NRVCs) from 2-day-old Sprague-Dawley rats were prepared as described [39]. For siRNA study, the oligonucleotides and transfection reagents were purchased from Thermo Fisher Scientific, Inc. The predesigned ON-TARGET plus SMART pool was used for knock-down of rat *HRC* gene. siRNA transfection was performed as described previously [40].

### SDS-PAGE and Western Blot Analysis

siRNA-transfected NRVCs or HL-1 cells were washed in cold PBS and lysed in lysis buffer containing 1% SDS, protease inhibitor cocktail (Roche Applied Science), 10 mM Tris-HCl (pH 7.4) and frozen hearts of shNC sham, shNC failure, shHRC sham and shHRC failure mice were grounded in a mortar and lysed in the same buffer. Cell lysates (60 µg) and heart homogenates (60 µg) were separated by electrophoresis on 6–15% gels and transferred to 0.2 µm nitrocellulose or 0.45 µm PVDF membranes. After blocking with 5% skim milk in 1× TBST, the membranes were incubated with respective antibodies: HRC, CSQ, TRN (home-made rabbit polyclonal sera); DHPR, NCX, CaMKII (Abcam); RyR2, SERCA2a, phospho-CaMKII and PLB (ABR); phospho-RyR2 and phospho-PLB-T17 (Badrilla); phospho-p38MAPK (Cell signaling); α-tubulin (Santa Cruz Biotech); phospho-PLB-S16 (Upstate). After primary antibody incubation, membranes were washed with 1× TBST and further incubated with proper peroxidase-conjugated secondary antibody. Western blot signal was detected by ImageQuant LAS 4000 mini (GE Healthcare Bio-Sciences AB) with a SuperSignal West Pico chemiluminescence kit (Thermo Fisher Scientific, Inc.). Western blot band intensities were measured by using ImageJ software.

### Calcium Transient Measurement

Ca^2+^ transients in NRVCs or HL-1 cells were measured as described previously [40]. Briefly, siRNA transfected NRVCs or HL-1 cells on glass coverslips were incubated with Fura-2 AM (Molecular Probes) in Tyrode solution containing 10 mM HEPES-NaOH, pH 7.4, 135 mM NaCl, 4.0 mM KCl, 1.0 mM MgCl_2_, 1.8 mM CaCl_2_, and 10 mM glucose for 30 min and washed in dye-free Tyrode solution. The cells were placed in a circulating bath with Tyrode solution held at 37°C under an inverted microscope. A dual-beam excitation spectrofluorometer setup (IONOPTIX) was used to record fluorescence emissions (505 nm) elicited from exciting wavelengths of 340 and 380 nm. Ca^2+^ transient amplitude measured as fluorescence ratio (340∶380 nm), cytosolic free Ca^2+^ concentration (baseline), time required to reach 50% of baseline (T_50_), and time to peak of Ca^2+^ transients were acquired. SR Ca^2+^ content was estimated by rapid application of 20 mM caffeine in Ca^2+^-free Tyrode solution. Data were analyzed by using Ion Wizard software (IONOPTIX). In the experiment shown in Figure 3, 1 µM isoproterenol (ISO) was used.

### Microsome (SR) Preparations from HL-1 Cells

Microsome preparations were carried out as described previously [41] with some modification. HL-1 cells grown on cell culture plate were washed with 5 ml of D-PBS (pH 7.4, WelGENE Inc., South Korea) containing protease inhibitor cocktail (Roche Applied Science) and harvested in the same solution by scraping. Cells were collected by centrifugation at 5500 rpm for 10 min in a Sorvall SS-34 rotor. Cell pellets were lysed with lysis buffer containing 0.6% phosphatydylcholine, 1 M Tris-HCl, pH 7.4, 0.1 M HEPES, 1 M NaCl, 1% CHAPS and 1X protease inhibitor cocktail, and incubated using rotatory incubator on 4°C. Lysed HL-1 cells were centrifuged at 5500 rpm for 10 min and the supernatant was centrifuged at 43000 rpm in a Beckman Ti-70 rotor for 60 minutes. The pellets were homogenized with storage buffer containing 1 M sucrose, 0.1 M HEPES, pH 7.4, 1 M KCl and 1X protease inhibitor cocktail in a Teflon-glass Dounce homogenizer and stored at −80°C. Protein concentration of the microsome was measured using BCA Protein Assay kit (Thermo Scientific).

### Tritium Ryanodine Binding Assay

[^3^H]ryanodine binding assay in NRVCs or HL-1 cells was performed as described previously [42], [43] with some modifications. Briefly, equilibrium ryanodine binding to whole homogenate (NRVCs) or SR (HL-1 cells) was measured by incubation of 0.05 mg of whole homogenate or SR in 250 *µ*l of reaction mixture containing 1 M KCl, 20 mM Mops (pH 7.4), various concentration of [^3^H]ryanodine and 50 *µ*M free Ca^2+^ for 2 hours at 37°C. PEG (poly-ethylene glycol) solution [100 *µ*l; 30% (w/v) PEG] was added to each vial and further incubated for 5 min at room temperature. Precipitated protein was segregated for 5 min at 12000 ***g*** in an Eppendorf microcentrifuge, and the pellets were rinsed twice with 0.4 ml of the washing buffer containing 1 M KCl, 20 mM Mops (pH 7.4) and 50 *µ*M free Ca^2+^. The pellets were then solubilized in 200 *µ*l of Soluene 350 (Packard, MA) at 70°C for 45 min and then radioactivity was counted with 4 ml of Picofluor (Packard) using a liquid scintillation counter (Beckman Coulter, Inc.). For nonspecific binding, a 1000-fold amount of non-radioactive ryanodine (Calbiochem, CA) was included. For the PEG solution, BSA and *γ* -globulin (5 mg of each per ml) were used as carrier proteins. [^3^H]ryanodine binding for NRVCs, whole homogenates were used.

### Oxalate-supported Ca^2+^ Uptake Assay

Ca^2+^ uptake rate was measured in siRNA transfected NRVCs or HL-1 cells as described previously [44]. Briefly, NRVCs or HL-1 cells were resuspended in the homogenization buffer containing 50 mM KH_2_PO_4_, 10 mM NaF, 1 mM EDTA, 0.3 M sucrose, 1X proteinase inhibitor cocktail and 0.5 mM DTT, and homogenized by Dounce glass homogenizer. 250 µg of cell lysates was added to 2.2 ml of the uptake buffer containing 100 mM KCl, 5 mM MgCl_2_, 5 mM NaN_3_, 0.5 mM EGTA, 1 µM ruthenium red, 200 µM CaCl_2_ and 40 mM imidazole, pH 7.0, and the reaction mixtures were incubated at 37°C for 4 min. The uptake reactions were initiated by serial addition of 5 mM K-oxalate and 5 mM Mg-ATP. Aliquots were filtered through a 0.45 µm Millipore filter after 1, 2, 3 and 4 minutes to terminate the reaction. The initial rate of Ca^2+^ uptake was calculated by linear regression analysis of the uptake values at 1, 2, 3 and 4 minutes. The results were analyzed using SigmaPlot 10 software.

### Transverse Aortic Banding

All animal protocols were approved by GIST Animal Care and Use Committee (the permit number: GIST-2011-1) and conform to the Guideline for the Care and Use of Laboratory Animals published by the United States National Institutes of Health. Male mice (C57BL/6) of 9 weeks old (23–25 g) were used for the present study. The animals were anesthetized with 0.3–0.5 ml of 1X Avertin solution (mixture of 2-2-2 tribromoethanol and tert-amyl alcohol) by intraperitoneal injection. Mice were ventilated with a tidal volume of 0.1 ml and a respiratory rate of 120 breaths per minute (Harvard Apparatus). A 2- to 3-mm longitudinal cut was made in the proximal portion of the sternum which allowed visualization of the aortic arch. The transverse aortic arch was ligated between the brachiocephalic and left common carotid arteries with an overlaying 27-gauge needle, and then the needle was immediately removed leaving a discrete region of constriction. One week post-operation mortality was less than 10%.

### AAV9 Production and Animal Injection

HEK-293T cells were obtained from the American Type Culture Collection (ATCC, Manassas, VA) and cultured in DMEM (Invitrogen) at 37°C and 5% CO_2_. All media were supplemented with 10% fetal bovine serum (Invitrogen) and 1% penicillin/streptomycin (Invitrogen). The recombinant viruses (rAAV9) were packaged as previously described [45], [46], [47]. Briefly, HEK-293T cells were plated on the Triple flasks (NUNC) and transfected with 50 µg of pds-AAV2 plasmid (which contain shNC: 5′-CTAGTTAGCGACTAAACACAT CAATTCAAGAGATTGATGTGTTTAGTCGCTAGTAT-3′, and shHRC: 5′-CTAGTCGATG TACCGA ATGTGAAATTCAAG AGATTTCACATTCGGTACATCGGTAT-3′) and 150 µg of pDG9 helper plasmid using the conventional CaCl_2_ transfection method. After 72 hours incubation, cells were harvested and collected with mild AAV lysis buffer (NaCl 150 mM, Tris 50 mM, pH 8.5). Cell lysates were produced by thawing and freezing method for 3 times. After incubation with DNase (Benzonase, Sigma) for 30 min, cell lysates were applied to the Iodixanol gradient (OptiPrep; Greiner Bio-One Inc.) to purify the viruses. Finally, viruses were concentrated using Vivaspin 20 Centrifugal concentrators 50K MWCO (Vivascience Inc.). Resulting rAAV viruses were titered via quantitative PCR to obtain DNase-resistant genome titers and stored at −80°C. Negative control (AAV9-shNC) and HRC-KD (AAV9-shHRC) viruses were injected into tail vein of 10-weeks-old C57 mice after 1 week of TAC and sham operation, amounting to approximately 10^10^ viral particles. Mice were sacrificed at 20-weeks-old and some hearts were prepared for trichrome staining and the others were stored at −80°C.

### 2D Guided M-mode Echocardiography

We performed two-dimensional (2D) guided M-mode echocardiography *in vivo* using a Toshiba PowerVision 6000 ultrasound system (Model SSA-370A, PLM-1204AT 12MHz-transducer) as previously described [48], [49], [50]. Briefly, mice were anesthetized with 0.3–0.5 ml of 1X Avertin solution (mixture of 2-2-2 tribromoethanol and tert-amyl alcohol) by intraperitoneal injection. Chests of the mice were shaved and echocardiography was performed. Diastolic and systolic left ventricle (LV) posterior wall thickness (PWTd and PWTs), LV end-diastolic dimension (LVEDD), LV end-systolic dimension (LVESD) and septal wall thickness (SWTd and STs) were measured.

### Histological Analysis of Hearts

Mice were euthanized by cervical dislocation under 2-2-2 tribromoethanol/tert-amyl alcohol anesthesia. Excised hearts were fixed in 4% formalin for 72 hours, embedded in paraffin, and sectioned (10 µm). Trichrome staining of the sectioned hearts was performed to measure the fibrotic areas. The fibrotic areas stained blue and the normal tissue stained red. Apoptosis was examined using the terminal deoxynucleotidyltransferase-mediated dUTP nick end labeling (TUNEL) assay kit (In Situ Cell Death Detection Kit, TMR red; Roche Applied Science) according to manufacturer’s instructions. TUNEL-positive nuclei in the heart section were calculated at X40 magnification under LSM-700 confocal microscope (Carl Zeiss Co., Ltd.).

### Statistics

All data are shown as mean ± SD. Statistical significance was analyzed by Student’s unpaired t-test. P<0.05 was considered statistically significant.

## Supporting Information

Figure S1 **siRNA-mediated HRC knock-down (KD) and expressional changes of SR proteins in HL-1 cells.** siNC and siHRC oligonucleotides (Dharmacon) were transfected to HL-1 cells. A: siRNA transfection efficiency in HL-1 cells. B: Western blot result of SR proteins after HRC-KD in HL-1 cells. RyR, ryanodine receptor; HRC, histidine-rich calcium binding protein; SERCA2a, sarcoplasmic reticulum Ca^2+^ ATPase 2a; DHPR, dihydropyridine receptor; NCX, Na^+^-Ca^2+^ exchanger; CSQ, calsequestrin; CaMKII, Ca^2+^/calmodulin-dependent kinase; TRN, triadin. C: Relative expression levels of SR proteins after HRC-KD. siNC, negative control of knock-down oligonucleotide; siHRC, HRC-KD oligonucleotide (**P<0.01). Note that there were no expressional changes of other SR proteins by knock-down of HRC.
(TIF)

Figure S2 **Depolarization- and caffeine-induced Ca^2+^ transients in HL-1 cells.** A: Typical records of Ca^2+^ transients in siNC and siHRC oligonucleotide treated HL-1 cells. B: Significantly changed parameters of Ca^2+^ transients after HRC-KD. Baseline, resting cytosolic Ca^2+^ concentration; peak amplitude, the amount of Ca^2+^ released from SR; T_50_, time to 50% baseline fluorescence; fractional Ca^2+^ release, depolarization-induced Ca^2+^ release/caffeine-induced Ca^2+^ release (*P<0.05).
(TIF)

Figure S3 **[^3^H]-ryanodine binding and oxalate-supported Ca^2+^ uptake assays in HL-1 cells.** After 48 h of siRNA transfection, the HL-1 cells were processed to [^3^H]-ryanodine binding and oxalate-supported Ca^2+^ uptake assays. A: [^3^H]-ryanodine binding assay results using siNC and siHRC transfected HL-1 cells. The significantly different B_max_ values of [^3^H]-ryanodine binding were 0.157±0.008 and 0.209±0.015 pmol/mg for siNC (○) and siHRC (•) samples, respectively. The Kd values are not significantly different between the 2 samples (siNC: 6.015±0.644 nM vs. siHRC: 5.314±0.654). B: Oxalate-supported Ca^2+^ uptake assay results. Oxalate-supported SR-based Ca^2+^ uptake in siNC (○) and siHRC (•) transfected HL-1 cells were determined at 1, 2, and 3 min. The rates of Ca^2+^ uptake were 19.29±2.62 and 26.14±1.42 nmol/mg/min for siNC and siHRC samples, respectively. Five sets of siHRC and 4 sets of siNC HL-1 cells were used for statistical analyses (*P value <0.05, **P value <0.01).
(TIF)

Figure S4 **AAV transduction efficacy in mouse heart.** DsRed was removed to enhance AAV-mediated knock-down (KD) efficiency of HRC in mouse heart. GFP-expressing AAV (AAV-GFP) was used to examine the transduction efficiency of our AAV systems. A: Western blot result showing AAV-mediated GFP expression in mouse heart. B: Fluorescence microscopic result for GFP in AAV-GFP transduced heart sample.
(TIF)
