# Supplementary figures and images for: AAV-Mediated Knock-Down of HRC Exacerbates Transverse Aorta Constriction-Induced Heart Failure
Source: PLoS One. 2012 Aug 28;7(8):e43282. doi: 10.1371/journal.pone.0043282 (PMC3429470; doi:10.1371/journal.pone.0043282)

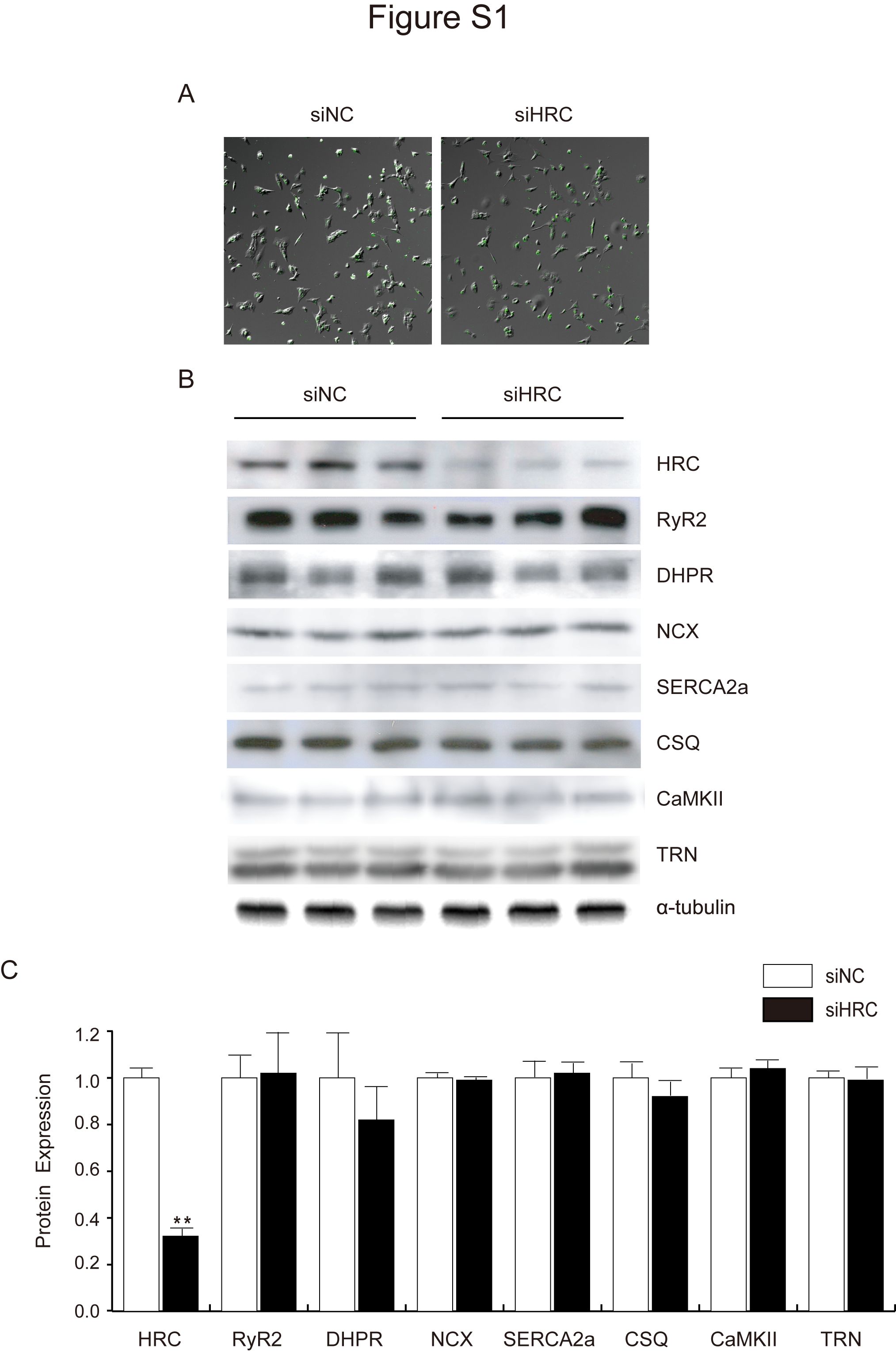

Supplement: Figure S1 — siRNA-mediated HRC knock-down (KD) and expressional changes of SR proteins in HL-1 cells. siNC and siHRC oligonucleotides (Dharmacon) were transfected to HL-1 cells. A: siRNA transfection efficiency in HL-1 cells. B: Western blot result of SR proteins after HRC-KD in HL-1 cells. RyR, ryanodine receptor; HRC, histidine-rich calcium binding protein; SERCA2a, sarcoplasmic reticulum Ca2+ ATPase 2a; DHPR, dihydropyridine receptor; NCX, Na+-Ca2+ exchanger; CSQ, calsequestrin; CaMKII, Ca2+/calmodulin-dependent kinase; TRN, triadin. C: Relative expression levels of SR proteins after HRC-KD. siNC, negative control of knock-down oligonucleotide; siHRC, HRC-KD oligonucleotide (**P<0.01). Note that there were no expressional changes of other SR proteins by knock-down of HRC. (TIF) [file pone.0043282.s001.tif]

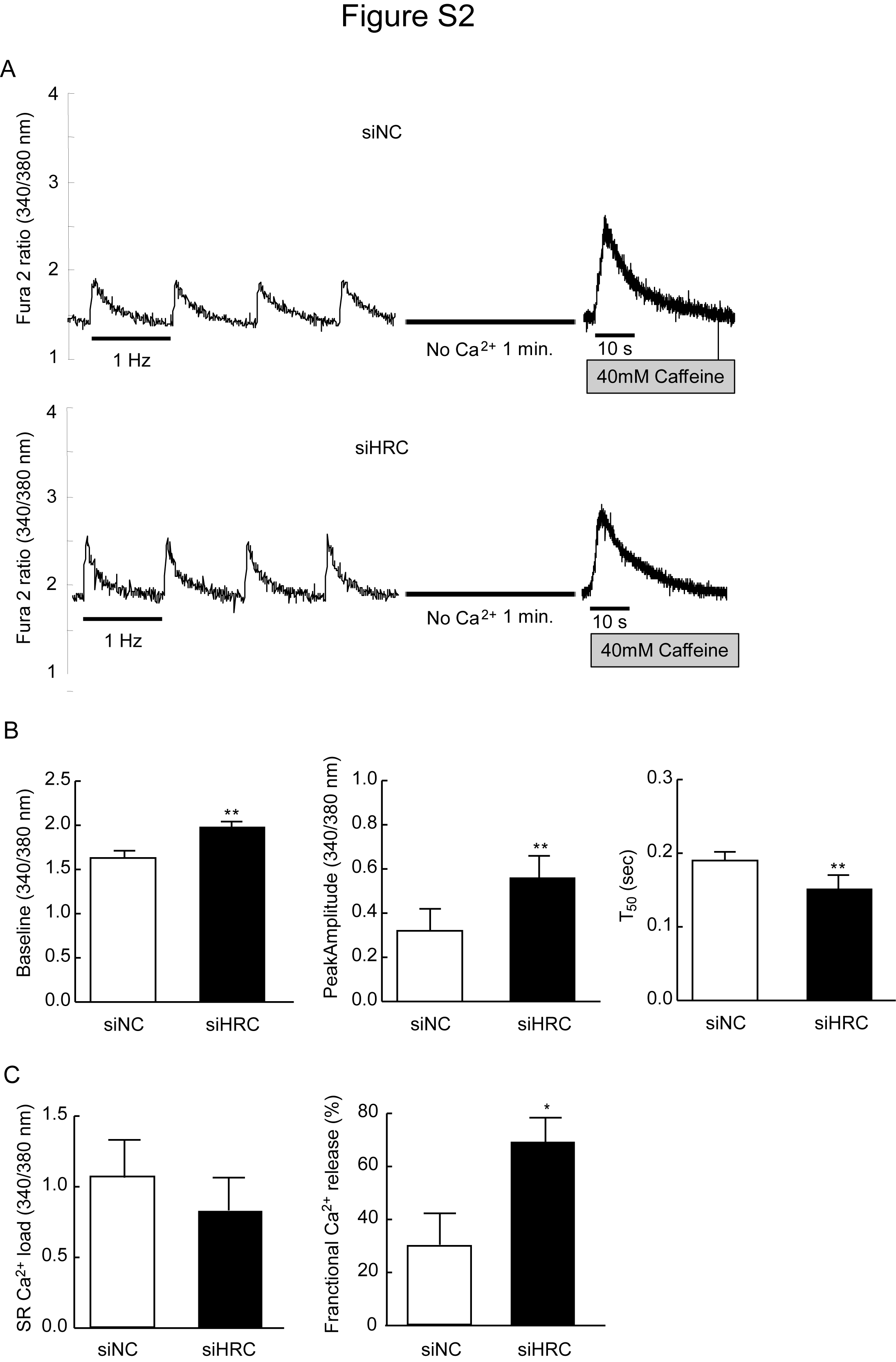

Supplement: Figure S2 — Depolarization- and caffeine-induced Ca2+ transients in HL-1 cells. A: Typical records of Ca2+ transients in siNC and siHRC oligonucleotide treated HL-1 cells. B: Significantly changed parameters of Ca2+ transients after HRC-KD. Baseline, resting cytosolic Ca2+ concentration; peak amplitude, the amount of Ca2+ released from SR; T50, time to 50% baseline fluorescence; fractional Ca2+ release, depolarization-induced Ca2+ release/caffeine-induced Ca2+ release (*P<0.05). (TIF) [file pone.0043282.s002.tif]

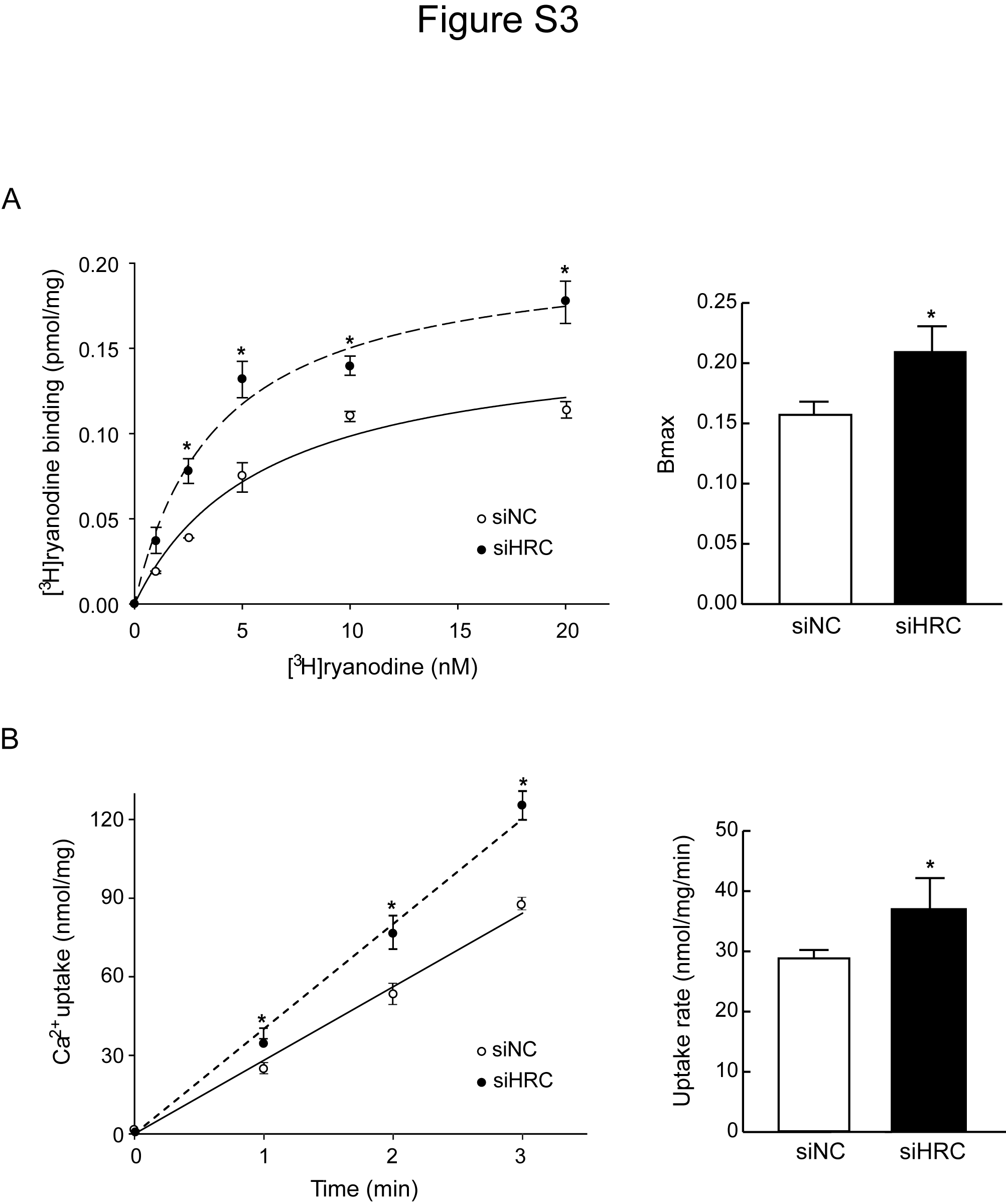

Supplement: Figure S3 — [3H]-ryanodine binding and oxalate-supported Ca2+ uptake assays in HL-1 cells. After 48 h of siRNA transfection, the HL-1 cells were processed to [3H]-ryanodine binding and oxalate-supported Ca2+ uptake assays. A: [3H]-ryanodine binding assay results using siNC and siHRC transfected HL-1 cells. The significantly different Bmax values of [3H]-ryanodine binding were 0.157±0.008 and 0.209±0.015 pmol/mg for siNC (○) and siHRC (•) samples, respectively. The Kd values are not significantly different between the 2 samples (siNC: 6.015±0.644 nM vs. siHRC: 5.314±0.654). B: Oxalate-supported Ca2+ uptake assay results. Oxalate-supported SR-based Ca2+ uptake in siNC (○) and siHRC (•) transfected HL-1 cells were determined at 1, 2, and 3 min. The rates of Ca2+ uptake were 19.29±2.62 and 26.14±1.42 nmol/mg/min for siNC and siHRC samples, respectively. Five sets of siHRC and 4 sets of siNC HL-1 cells were used for statistical analyses (*P value <0.05, **P value <0.01). (TIF) [file pone.0043282.s003.tif]

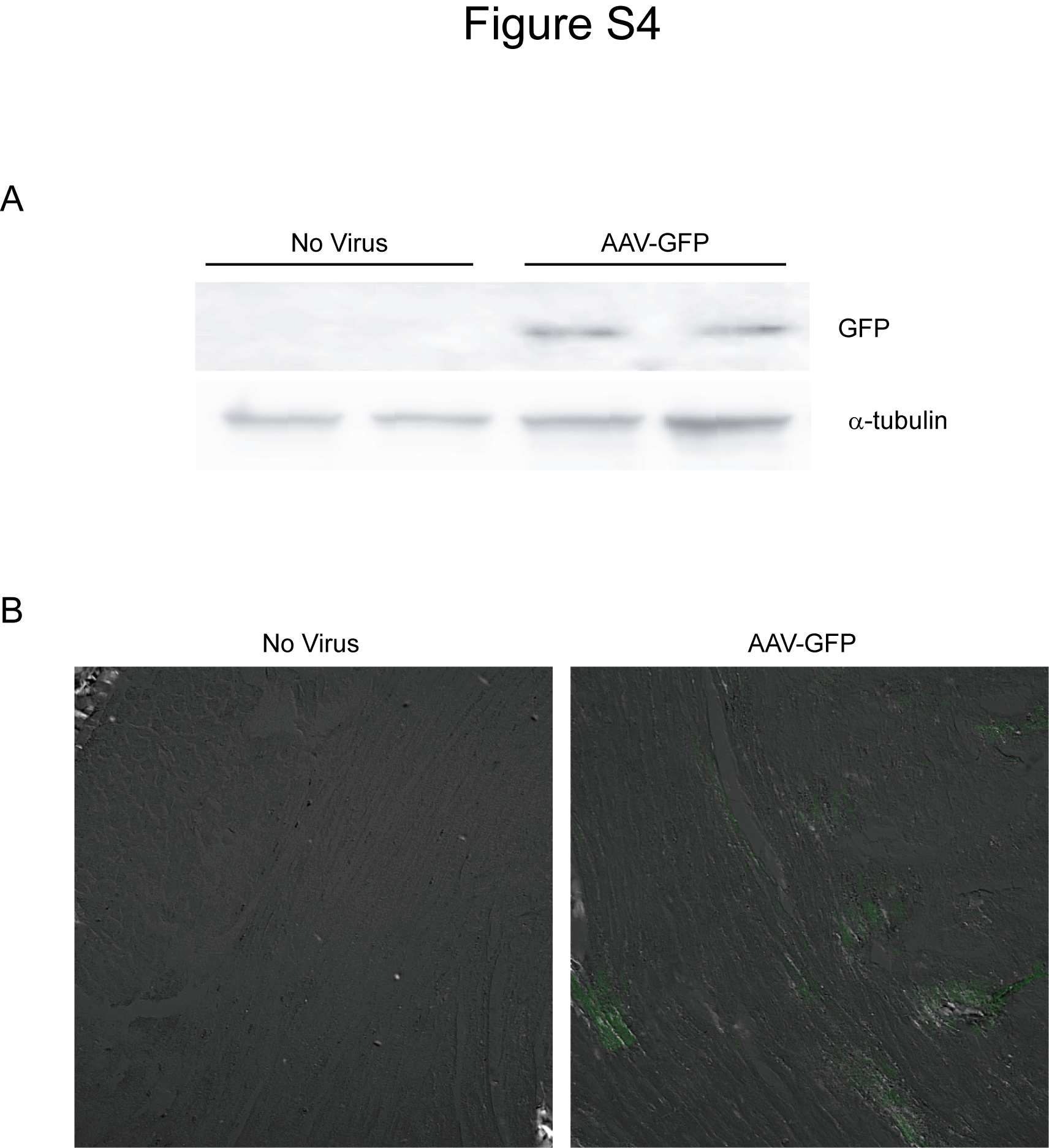

Supplement: Figure S4 — AAV transduction efficacy in mouse heart. DsRed was removed to enhance AAV-mediated knock-down (KD) efficiency of HRC in mouse heart. GFP-expressing AAV (AAV-GFP) was used to examine the transduction efficiency of our AAV systems. A: Western blot result showing AAV-mediated GFP expression in mouse heart. B: Fluorescence microscopic result for GFP in AAV-GFP transduced heart sample. (TIF) [file pone.0043282.s004.tif]
